# Comparative Quantitative Mass Spectrometry Analysis of MHC Class II-Associated Peptides Reveals a Role of GILT in Formation of Self-Peptide Repertoire

**DOI:** 10.1371/journal.pone.0010599

**Published:** 2010-05-12

**Authors:** Branka Bogunovic, Priya Srinivasan, Yumi Ueda, York Tomita, Maja Maric

**Affiliations:** 1 Department of Microbiology and Immunology, Georgetown University School of Medicine, Washington, D. C., United States of America; 2 Lombardi Comprehensive Cancer Center, Georgetown University School of Medicine, Washington, D. C., United States of America; New York University, United States of America

## Abstract

Gamma interferon Inducible Lysosomal Thiol reductase (GILT) is a unique lysosomal reductase that reduces disulfide bonds of endocytosed proteins. Lack of GILT clearly decreases CD4 T cell-antigen specific responses against some epitopes of antigens containing disulfide bonds, but not to proteins with few or no disulfide bridges. Hence, global impact of GILT on antigen presentation is currently not well understood. We used Nano-LC-ESI-MS/MS to investigate how GILT affects diversity of self-peptides presented by MHC class II molecules. Surprisingly, the repertoire of self-peptides in the absence of GILT does not appear to be significantly different, as only few peptide species (∼2%) were found to be the unique indicators of GILT's presence or absence. In the absence of GILT about thirty peptide species (∼5%) were found either uniquely or fourteen to hundred fold more abundantly expressed than in the presence of GILT. Our data indicate that GILT has limited yet unexpected effect on self-peptide species presented by MHC class II antigens.

## Introduction

Self-peptide/MHC complexes have several important roles in the physiology of T cells. Engagement of the T-cell receptors with self-peptide/MHC complex is the basis for TCR repertoire formation in the thymus and thymocyte maturation [1]. Homeostasis of naïve T cells and maintenance of functional competence of memory T cells in the periphery depends on the constant engagement with self-peptide/MHC complexes [2]. In addition, it is thought that self-peptide/MHC complexes may modulate the strength of the immune responses to foreign antigens [3]. Thus, alteration in processing of self-proteins may yield peptides with different immunogenicity, thereby it may be responsible for (or take part in) autoinflammation and responses to cancer. Therefore it is important to identify and understand factors that influence diversity of presented self-peptides.

Self-peptides presented by MHC class II molecules are generated in specialized endocytic compartment with acidic pH. Proteins imported to endosomes are first denatured by action of GILT and further processed into peptides by cathepsins. Peptides generated by enzymatic processing are loaded onto MHC class II and exported to the cell surface. We have previously shown that GILT plays an important role in processing and presentation of exogenous proteins [4]. Our studies indicated that GILT is involved in denaturation of proteins containing at least four disulfide bridges, such as: Hen Egg Lysozyme (HEL), RNAse A, insulin. Further studies indicated that proteins that contain no disulfide bridges, for example bovine alpha casein, do not require GILT for processing. However, epitopes within the same protein containing multiple disulfide bridges, such as HEL, are differentially affected by the absence of GILT. Because of this, the effect of GILT on global antigen processing is not readily predictable.

Here, we studied the effect of GILT on the expression of self-peptides in the context of MHC class II. We hypothesized that due to lack of GILT, processing of self-proteins will be altered in such a manner that the number and/or the quantities of self-epitopes would be diminished/reduced relative to GILT wild type (WT) cells. To our surprise, mass spec analysis revealed more abundant presentation of most self-peptides in the absence of GILT, and even appearance of 10 novel epitopes. This finding suggests that processing of a number of proteins is enhanced in GILT−/− splenocytes, which is in apparent contradiction to previous observation [4] that GILT−/− splenocytes process certain epitopes from exogenous antigens less efficiently than the WT cells.

Among proteins identified as the source of MHC class II associated peptides either exclusively, or fifty or more fold expressed in GILT−/− cells more than in GILT-WT, are proteins involved in apoptosis, mitosis regulation and transcription factors. We have also validated a binding of a limited number of peptides found to bind exclusively to GILT−/− derived MHC class II. Therefore, our data indicate that self-proteins involved in some of the fundamental cellular processes might be processed differently in the absence of GILT and presented on the cell surface more frequently.

## Results

### Isolation of MHCclass II/peptide complexes from GILT−/− and GILT-WT mouse splenocytes

**Figure 1:**
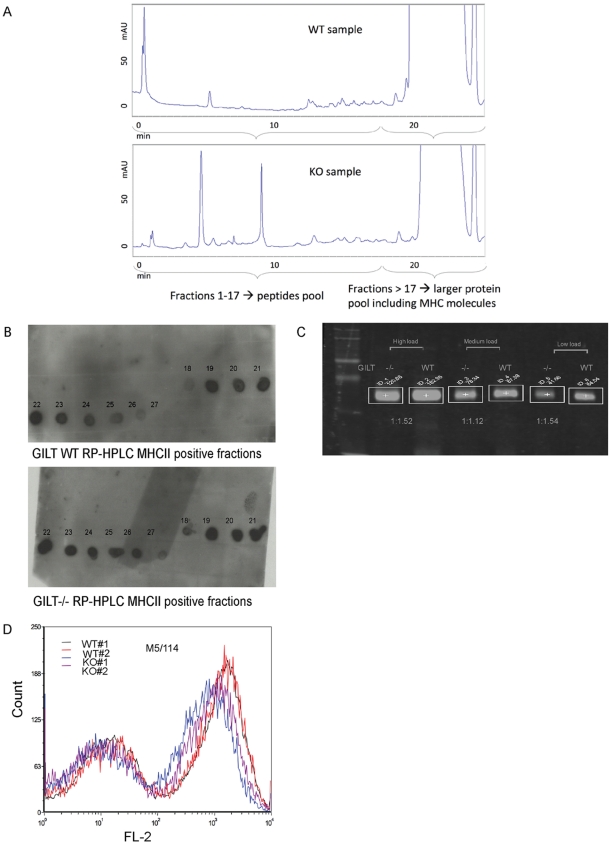
Purification of MHC class II-associated peptides. **A. RP-HPLC chromatogram of elution of the MHCII associated peptides.** System: Agilent 1100, Column: Agilent ZORBAX 300SB-C18, Flow rate : 1 mL/min and a gradient was created by mixing Solvent A: 0.1% TFA (v/v) in CH3COOH(8.7%) and HCOOH(2.2%), pH1.9, and Solvent B: 0.08% TFA (v/v) in Acetonitrile; **B. Dot blot of RP-HPLC fractions.** 5 µL from each fraction was applied to a blotting membrane and I-A^b^ molecules were detected as described in the text. **C. Quantitative Imaging Analysis of MHCII positive fractions from GILT-WT and GILT−/− samples.** MHC class II positive fractions from RP-HPLC were pooled for each sample and three different amounts (high: 25 µL, medium: 12.5 µL, and low: 6µL) from each pool were loaded onto SDS-PAGE gel for quantitative imaging. **D. Flow cytometry analysis of GILT−/− and GILT-WT splenocytes.** Spleens were isolated, ground and red blood cells lysed in hypertonic buffer. Washed and filtered splenocytes were incubated 30 min. on ice with anti-IA^b^ M5/114–PE antibody.

To isolate MHC class II-bound peptide complexes, MHC class II I-A^b^ molecules were purified from spleen cells derived from GILT-WT and GILT−/− C57BL/6 mice. NP40 cell lysates were subjected to affinity chromatography using I-A^b^-specific monoclonal antibody Y3jP. The MHC class II-associated peptide fractions were eluted with the DEA buffer and purified by RP-HPLC, which doubled as acid elution of peptides from IA^b^ molecules (Fig. 1a).

Dot blot analysis (Fig. 1b) was performed to test each fraction for the presence of I-A^b^. All the I-A^b^-positive fractions were combined to determine the ratio of the total I-A^b^ protein amounts between the GILT−/− and the GILT-WT samples. Quantitative immunoblot analysis by Odyssey showed that the GILT−/−/GILT-WT I-A^b^ ratio was 1/1.5 (Fig. 1c). Flow cytometry analysis of splenocytes with anti-IA^b^ antibody M5/114 show that GILT−/− splenocytes express mildly lower level of IA^b^ (Fig 1d).

The I-A^b^-negative fractions containing short peptides (mostly MHCII-associated peptides released from MHCII) from each sample were pooled and prepared for iTRAQ labeling. Two different iTRAQ reagents were used to label GILT−/− and GILT-WT samples separately. Once labeled, the two samples were combined for the rest of the procedures to ensure equal treatments to the samples.

### More abundant self-peptide presentation in the absence of GILT

The labeled samples were further separated with ion-exchange and nano reverse phase (RP) HPLC chromatography and nano RP-HPLC was directly interfaced with Q-TOF mass spectrometer through Electrospray Ionization (ESI). A large amount of data collected from the mass spectrometer was rapidly processed by analysis software Protein PILOT with the search engine PARAGON [5] to identify peptides sequences of I-A^b^-bound peptides which resulted in the detection of more than 5,000 peptide species. Of these, 511 distinct peptides with iTRAQ117 (GILT−/− sample) and/or iTRAQ114 (WT sample) were considered statistically significant based on the confidence score associated with the peptide identification. These peptides are listed in Table S1. Relative abundance of each peptide was determined by the ratio of iTRAQ117 and iTRAQ114 signal intensities, corrected by the factor of 1.5, reflecting the ratio of MHC class II molecules in the two samples based on the quantitative immunoblot analysis, Fig 1c. 5.5% among these peptides (28 out of 511) were more abundant in GILT-WT samples, while the remaining 94.5% were more abundant in GILT−/− sample. Only 2.0% of these (10 out of 511) were detected exclusively in GILT−/− samples (Table 1), and 3.5% of these peptide species (18 out of 511) were approximately 10 to 60 fold more abundant in GILT−/− samples (Table S2). This is in contrast to WT sample, where no unique peptides were detected and the peptide was only six fold more abundant at most. The peptides found exclusively in GILT−/− fractions (Table 1) originate from proteins involved in cell division, apoptosis and development, while one has unknown function (2610002D18Rik protein).

**Table 1:** Peptides uniquely expressed by MHC class II isolated from GILT−/− cells.

Peptide sequence	Length (aa)	Peptide Source	Position*	No of Cys**
ASSPAVTAP	9	Q9QY61 Iroquois homeodomain protein IRX-4 (515aa)	408–416	10
KLDLGSGGRALGGVGTAPAGGPAS iTRAQ4plex(K)@1	24	BAF64834.1 Apoptosis-associated Tyr kinase 3 (1424aa)	1080–1103	18
SPRIQLSSCRSLESLR Protein Terminal iTRAQ4plex@N-term, Methylthio(C)@9	16	Q8BKW6 Putative uncharectarized protein (Fragment) [Syde 2] (1096aa)	1–16	27
VGAVAAAEAAGLPGGGEGPKLAEG iTRAQ4plex(K)20	24	AAI50821.1 Magi1 protein (1280aa)	44–67	17
VGNVENTLFINTSNHGVF Oxidation(N)@3	18	O70472 Transmembrane protein 131 (Protein RW1) [Tmem131] (1829aa)	130–147	12
GGAGGGGAGGGAGGGRSPVRELDM Deamidated(R)@20, Oxidation(M)@24	24	P70390 Short stature homeobox protein 2 (Homeobox protein Og12X) (OG-12) (Paired family homeodomain protein Prx3) [Shox2] (331aa)	91–99	4
LTMMSLTQPVLMGT Deamidated(Q)@8	15	B2RR86 2610002D18Rik protein [2610002D18Rik] (339aa)	138–152	9
GGGGGGFGGSGG	12	NP031868.2 DEAH (Asp-Glu-Ala-His) box polypeptide 9 (1383aa)	1253–1264	23
SGQEADSE	8	Q8C0D9 Centrosomal protein of 68 kDa (Cep68) [Cep68] (733aa)	15–92	12
TLGTGKGT iTRAQ4PLEX(K)@6	8	P24860 G2/mitotic-specific cyclin-B1 [Ccnb1] (430aa)	68–75	5

*Position of peptide within protein.

**Number of cysteines in protein that was identified as a source of peptide.

The lengths of the MHCII-associated peptides from the GILT−/− sample ranged from 8 to 24 amino acids, thus not showing any preferential binding for the peptides of certain length. Only one of the ten peptides found exclusively in the GILT−/− sample contained a single cysteine (SPRIQLSSCRSLESLR), while proteins that this group of peptides originated from, all contain multiple cysteines from just four to up to 28. All peptides except two were positioned either at the very N or C-termini of the respective proteins. Physical location of the peptide within the native protein leads to differential antigen processing and consequent epitope selection [6]. It is possible that in the folding pathway of the protein, these regions are probably among the last to become structured, and they would consequently be easily unfolded and preferentially accessible to fragmentation by the proteases in the antigen-processing pathway in the absence of GILT. Unfortunately, crystal structures are not yet available for any of these proteins of mouse origin therefore we can only speculate that these epitopes are perhaps positioned in such a manner in a respective protein that either protein denaturation facilitated by GILT is not necessary or alternative processing takes place that is independent of GILT. Similarly, among the peptides present in excess (at ten to sixty fold) in MHC class II isolated from GILT−/− samples, only two out of 18 peptides contained cysteine residues. However, all 18 peptides originate from the proteins that contain at least one or more cysteine in their primary sequence. Therefore, overall almost 90% of epitopes presented exclusively or abundantly by MHC class II from GILT−/− samples are cysteine-free although all originate from proteins that contain cysteines.

Together these data indicate that there is an altered and preferential expression of self-peptides on the surface of GILT-deficient cells. Therefore, our data support the hypothesis that the absence of GILT alters the presentation of MHC class II associated self-antigens, especially for the protein containing a large number of cysteine residues. However, it is somewhat surprising that the absence of GILT dramatically increases both quantity and to a limited degree the diversity of presented peptides.

### Detection of Peptide modifications

ProteinPILOT search engine was able to detect various peptide modifications: oxidation (Met), deamidation (Asn and Gln), phosphorylation (Ser, Thr, Tyr), acetylation (N-terminus), methylthionilation and N-terminal pyroglutamate and sulfation. Many peptides have multiple posttranslational modifications. About 60% of the peptides (285 out of 511 peptides) were found to have modifications, which is in a good agreement with the current estimation that more than 50% of proteins are modified in the physiological environment. Among the most frequent modifications found were oxidation and deamidation. The amino acids most susceptible to oxidation include methionine, cysteine, histidine, and tryptophan, and the products formed from oxidation of these residues have predictable shifts in molecular weights compared to the molecular weights of unoxidized structures. Because oxidation and deamidation can occur during sample preparation or during HPLC or mass spectrometric analysis, it can complicate interpretation [7], [8], thus these modifications are not included in Table 2. However, other identified posttranslational modifications require the activity of enzymes and are less likely to be an artifact of sample handling. Samples were treated with the mix of protease inhibitors and were kept at 4°C at all times. Analysis of data from Table 2 reveals that there is no significant difference in percentage of modified peptides among the peptides that are predominantly expressed in either GILT-WT (28.6%) or GILT−/− (19.2%) samples. Low percentages of peptides predominantly expressed by MHCII from GILT−/− sample are phosphorylated (2.27%), Glu to Pyro-Glu (1.86%), Gln-Pyro-Glu (0.6%) and dethiomethyl modified (0.41%) while peptides predominantly expressed in GILT-WT lack these modifications. However, higher percentage of peptides more abundantly expressed in GILT-WT, show N-terminus acetylation (7.1% vs. 1.24%) and Pro to Pyro-Glu (10.7% vs. 0%).

**Table 2:** List of Peptide modifications.

Type of Modification	117∶114<1	117∶114>1	117∶114<0.5	117∶114>2
Total no. of peptides 511	28	483	5	341
Methylthio (C)	3 (10.7%)	45(9.3%)	0	31
Methylthio @N-term	0	3(0.62%)	0	2
Phosphorylation (S or T)	0	11(2.27%)	0	9
Protein terminal acetyl @N-term	2(7.1%)	6(1.24%)	1	1
Pro->Pyro-Glu	3(10.7%)	0	0	0
Glu-> Pyro-Glu	0	9(1.86%)	0	7
Gln->Pyro-Glu	0	3(0.6%)	0	3
Dethiomethyl	0	2(0.41%)	0	1
Dehydrated (all aa)	1(3.57%)	6(1.24%)	0	5
Total peptides modified	8(28.6%)	93(19.2%)	1	63

Total number of peptides labeled with both iTRAQ117 and iTRAQ114 labels is 511. 28 of these peptides were more abundantly expressed in MHCII from GILT-WT sample (labeled with iTRAQ114) <1, and 483 peptides were more abundant in GILT−/− samples. The amount of MHC class II from GILT-WT and GILT−/− sample was normalized based on quantitative W. blot of IA^b^.

### Validation of peptide binding

Mass spectrometry analysis of GILT−/− and GILT-WT samples was accomplished twice with similar results. However, to confirm that at least some of identified peptides can bind to IA^b^ molecules in vitro, we selected six peptides from the peptides expressed either exclusively or at least ten fold more abundantly in the GILT−/− samples (Table 1 a, b) than in GILT-WT to test their binding to MHC molecules in an *in vitro* assay. We used web-based Rankpep software [9] to predict the affinities of 9 amino acid IA^b^-binding core of each peptide (Table 3). RANKPEP uses Position Specific Scoring Matrices (PSSMs) or profiles from set aligned peptides known to bind to a given MHC molecule as the predictor of MHC-peptide binding and reports scores (sum of the profile scores that match the residue type and position in the profile) and percentile scores relative to the optimal sequence.

**Table 3:** Peptides used for validation of peptide binding to IA^b^.

Peptide sequence	N-term	MBS	C-term	Length	Peptide source	Rankprep score	%OPT
1. * ASFEAQGALANIAVDKA	SFE	AGQGALANIA	VDK	17aa	Ea 56–73**	10.439	29.30%
2. * CASPLITTATFAYWGQGT		CASPLITTA	TFA	18aa	AAX90134.1| immunoglobulin heavy chain (24aa) FRAGMENT	13.912	39.04%
3. * SAQVVVGPVSEAEPPKASSA	VGP	VSEAAEPPKA	SSA	20aa	Q9D424 Calcium-binding tyrosine phosphorylation-regulated protein (Calcium-binding protein 86) (453aa)	11.814	33.16%
4. * SGAQPGGVPSAPTGPLGPP	GGV	PSAPTGPLG	PP	19aa	Q9Z1R2 Large proline-rich protein BAT3 (1154 aa)	7.485	21.01%
5. ASSPAVTAP		ASSPAVTAP		9aa	Q9QY61 Iroquois homeodomain protein IRX-4 (515aa)	7.092	19.90%
6. ATAGSGGVNGG	A	TAGSGGVNG	G	11aa	Q00PI9 Heterogeneous nuclear ribonucleoprotein U-like protein 2 (MLF1-associated nuclear protein) [Hnrnpul2] (745aa)	0.756	2.12%
7. AATEGTTAT		AATEGTTAT		9aa	Q6PGB8 Probable global transcription activator SNF2L1 (Nucleosome-remodeling factor subunit SNF2L) (ATP-dependent helicase SMARCA1) (1046aa)	−1.538	−4.32%
8. *** RTYTYEKL				8aa		N/A	N/A
9. *** VGYMYETL				8aa		N/A	N/A

*Actual synthetic peptides used to validate binding to IA^b^ are in black.

**Eα 56–73 [39].

***Negative control peptides that bind to H2-K^b^ [12].

Matrix:I_A^b^.p.mtx, Consensus: YYAPWCNNA, Optimal score 35.632, Binding threshold: 9.52.

Rank: Relative rank of predicted peptide (position specific scoring matrices (PSSM)).

%OPT -% score of the predicted peptide relative to that of consensus. The consensus is the sequence that yields max score.

MBS-Minimal Binding Sequence.

**Figure 2:**
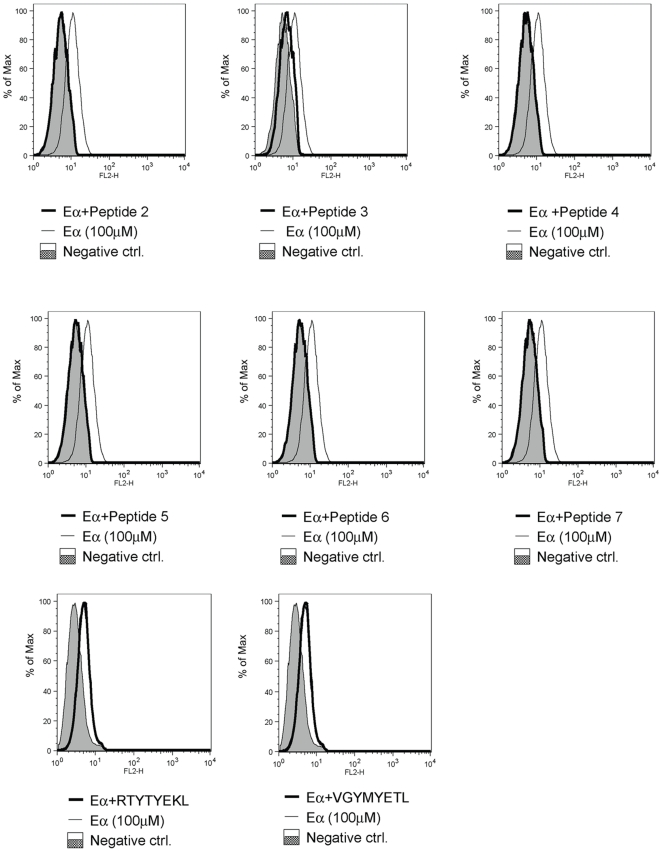
Binding of peptides to IA^b^. T2.IA^b^ cells were incubated for 1h at 37°C with either Eα peptide 56–73 (ASFEAQGALANIAVDKA) alone, or with mix of Eα peptide and individual peptides 2 to 6 listed in Table 3. Peptide Eα was used at 100 µM while competitor peptides 2–6 were used at concentration of 2.5 mM. Upon incubation with peptide mix, cells were extensively washed in PBS and the binding of Eα 56–73 was detected by incubation with YAe (anti-IA^b^) antibody and secondary antibody conjugated with PE. Cells incubated only with YAe antibody and secondary antibody without any peptide were used as a negative control. Presence of any of the competitor peptides 2–6, decreased the binding of Eα 56–73 signifying the binding of peptides to IA^b^ and outcompeting the binding of Eα 56–73. Two peptides RTYTYEKL and VGYMYETL known to bind to H2-Kb [12] served as negative control for specificity of binding.

To further solidify the evidence, we used T2.IA^b^ cell line as antigen presenting cell in peptide competition binding assay between a known strong antigen and the peptides we found in our study. Binding of peptides to IA^b^ was determined by flow cytometry. T2.IA^b^ cells were incubated with Eα peptide 56–73 (ASFEAQGALANIAVDKA) known to bind to IA^b^ with a high affinity [10]. Eα peptide bound to IA^b^ is specifically recognized by Y-Ae antibody [11]. Peptides used for competition with Eα for binding to IA^b^ are shown in Table 3. T2.IA^b^ cells were incubated with either synthetic Eα 56–73 alone or Eα 56–73 in combination with each one of the peptides from Table 3 for 1hr at 37°C. Incubation of peptides at 4°C yielded identical results (data not shown), however incubation at 37°C is mimics more closely physiological conditions. Cells were then washed and incubated with Y-Ae antibody and secondary antibody conjugated with PE. Figure 2 shows that of six chosen peptides five peptides fully diminish the binding of Y-Ae antibody and the sixth one had a partial effect. This result suggests that chosen peptides were able to out compete the binding of Eα 56–73 to IA^b^ and therefore validates the results of our mass spec analysis. As a control for specificity of binding to IA^b^ we used two peptides that bind to H2K^b^: RTYTYEKL and VGYMYETL [12]. As expected, neither of these peptides was able to out compete Eα binding.

## Discussion

Absence of lysosomal thiol reductase GILT can affect immune responses to viral [13], [14], tumor [15], [16], and parasite antigens (Dr. N. Nanda and Dr. M. Maric, unpublished), and can affect the development of autoimmune diseases, such as experimental allergic encephalomyelitis (Dr. N. Ruddle, unpublished data) and diabetes [17]. The major mechanism is thought to be deficient antigen processing and presentation in the absence of GILT, as suggested by experiments using model antigens, where GILT was required to reduce disulfide bonds of endocytosed proteins leading to protein unfolding [4]. Reduced forms of proteins are more amenable to further processing by lysosomal proteases into peptides that eventually bind to MHC class II molecules.

Present findings thus appear at odds with an expectation that lack of GILT might lead to fewer antigens being presented by MHC class II molecules. It should be pointed out, however, that our current analysis evaluates steady state of processing and presentation of permanently present self-antigens, whereas previous studies have assessed the ability of antigen processing machinery to generate epitopes from antigens introduced in the system at will (exogenously and transiently). It is therefore possible that differences in the non-self epitope presentations in the presence or absence of GILT would have been reduced, or perhaps annulated, if foreign proteins were expressed permanently in the experimental system.

The above reasoning could explain findings of relatively equal self-peptide representation in the absence or presence of GILT. However, present data suggest overall more abundant peptide presentation in the absence of GILT. It is possible that GILT may influence activity of other enzymes or events involved in self-protein processing. We have shown previously that oxidative stress is increased in GILT−/− cells [18]. Oxidative stress is known to increase autophagy [19], [20] and therefore, we can speculate that increased autophagy in GILT−/− cells lead to increased variety and quantity of proteins exposed to lysosomal enzymatic processing. Therefore, larger amount of certain peptides, and new peptide species maybe expressed by MHC class II molecules at the cell surface. This however remains to be tested in the future.

MHC class II-associated self-peptides are derived from proteins that normally do not intersect the endocytic compartment and are usually presented by the MHC class I pathway. However, previous studies have shown that there is an extensive antigenic “cross-talk” between the classical MHC class I and class II presentation pathways in professional APCs. For example, macrophages and DCs efficiently perform MHC class I-mediated presentation of exogenous protein antigens internalized by macropinocytosis or phagocytosis [21], [22], [23]. Furthermore, Dongre, *et al.* demonstrated examples of MHC class II-mediated presentation of self–peptides [24]. These self-peptides could potentially gain access to endosomal/lysosomal compartments through apoptosis, necrosis, and/or autophagy. Therefore, in at least some cases self-proteins may become exposed to the GILT-positive compartment and their processing might be affected by GILT.

ESI-MS/MS was originally applied to the analysis of MHC class I and MHC class II-associated peptides by Hunt, *et al.*, where 650–2,000 different peptides bound to murine class II allele, I-A^d^ were discovered [25]. However, peptide identification was limited due to slow tandem mass spectral data acquisition, as well as manual interpretation of peptide sequences. More recently Dongre, *et al.*, applied rapid, high throughput MHC-bound peptide sequence analysis. Improved sample preparation was combined with automated tandem mass-spectral data acquisition and computer-assisted interpretation of tandem mass spectra [24]. The SEQUEST computer algorithm was used to compare experimental and theoretically generated mass spectra. Using this strategy, they characterized 128 mouse MHC class II (I-A^b^) associated peptides presented on the surface of B cells and macrophages. We further improved the method for isolation of MHC class II-associated peptides. Dongre, *et al.*, used 2.5 M acetic acid to separate peptides from MHC class II by ultracentrifugation through a 10-kDa cut-off Amicon filter, prior to RP-HPLC fractionation. This approach could potentially lead to a loss of a portion of the sample due to the property of Amicon ultracentrifugation membrane. Instead, we directly applied the MHC class II-bound peptide mixture to the RP-HPLC column at the acidic condition (0.1% TFA, 8.7% acetic acid (1.5M) and 2.2% formic acid (0.58M) in water) to achieve acid dissociation of the peptides from MHC class II during the purification. The following gradient step with acetonitrile separated short peptides from MHC class II and other contaminants. In order to efficiently analyze samples containing a large number peptides we used multi-dimensional liquid chromatography together with automated tandem mass spectral data acquisition on a QSTAR ELITE Hybrid LC/MS/MS, with the latest Analyst® QS 2.0 software.

A large MS/MS dataset is valuable only if it can be efficiently processed to identify peptide sequences. Comprehensive analysis of peptides by the ProteinPILOT software with the Paragon database search algorithm increased the number of peptides found, improved sequence coverage, and increased peptide identification (www.appliedbiosystems.com). Increasing the search space by considering many more biological modifications and imperfect enzymatic cleavages such as: Carboxamidomethyl Cys, Deamidation, Oxidation (M), PyroGlutamic Acid (Q), O-Phosphoryl S/T/Y, Protein-N-Terminal Acetyl, allowed the identification of significantly increased number of peptides in our study in comparison to the similar studies performed in the past. Together, these modifications in the peptide purification method and data acquisition, allowed improvements in the quality and quantity of the identified peptide sequences. Comparison of modifications in peptides predominantly expressed in GILT-WT sample vs. GILT−/− sample did not show statistically significant differences due to relatively small numbers of modified peptides (8 modified peptides out of 28 predominantly expressed in GILT-WT, and 93 modified out of 483 predominantly expressed in GILT−/− sample). However, several modifications were detected exclusively in peptides from GILT−/− sample: phosphorylation, methylthio N-term, Glu and Gln to pyro-Glu and dethiomethyl. This raises a possibility that GILT influences directly or indirectly posttranslational modifications of certain proteins, but this remains to be explored further in another study.

It is estimated that between 50 and 90% of proteins in human body are modified posttranslationally. Protein and peptide modifications have been shown to influence the immunogenicity of peptides and proteins presented by MHC molecules [26], [27], [28], either by creating neo antigens or masking antigens normally recognized by the immune system. Therefore, peptide modification plays important role in the autoimmune response as well as in anti-tumor response and/or can be used as a strategy by pathogens for their survival in the host. Several studies have shown that posttranslational modification of self-peptides can induce autoimmune response. For example the acetylated N-terminal peptide of myelin basic protein is found to be required for development of experimental autoimmune encephalomyelitis (EAE) - a murine model of human multiple sclerosis. Nonacetylated peptide fails to stimulate T cells and will not elicit EAE [29]. Other examples are the findings that T cell responses specific for phosphorylated αB–crystalline exist in EAE [30], while phosphorylation of multiple antigens in Systemic Lupus Erythematosus (SLE) induces diverse B cell response [31], [32], and deamidation of wheat gliadin induces specific T and B cell responses in celiac disease in humans [33], [34]. It has been shown also that for MHC class I associated peptides deamidation, cysteinilation and phosphorylation shape anti-tumor CTL response [35], [36], [37]. In addition, reduction of cysteine containing epitopes of nucleoprotein of influenza increases their immunogenicity 10–100 fold [28]. Therefore, it is possible that presence of different peptide modifications in the absence of GILT may also contribute to altered immune responses that were previously studied [4], [17].

In summary, labeling the GILT-WT sample with the iTRAQ isotope tag-114, and the GILT−/−sample with the iTRAQ isotope tag-117 allowed comparison of each identified peptide. Quantitative data analysis based on the GILT KO/WT (114∶117) peptide ratio suggested overall increase of peptide amounts in the GILT−/− sample in comparison to the GILT-WT sample. In addition, we identified a small subset of ten peptides exclusively presented by MHC class II in the absence of GILT and another subset of 18 peptides that are present in the GILT−/− MHCII pool 10 to 60 fold more abundantly than in the GILT-WT MHCII pool. These findings suggest that the diversity of the MHC class II-bound self-peptides presentation is only moderately affected by the absence of GILT and that few neoepitopes emerge from self-proteins. Together these findings support the hypothesis that the absence of GILT alters the presentation of self-antigens on the cell surface.

## Materials and Methods

### Mice, cells and antibodies

Spleens of one hundred C57BL/6.GILT-WT and one hundred C57BL/6.GILT−/− mice (8–12 weeks old) were used as a source of the cells. Spleens were ground in PBS, pH 7.4 and red blood cells were removed by lysis in ACK lysis buffer (150 mM NH_4_Cl. 10 mM KHCO_3_. 0.1 mM Na_2_EDTA). Splenocytes (5×10^9^) were lysed in PBS, 1% NP-40, 20mM PMSF, 10mM TLCK, and 25 mM iodoacetamide, pH 7.4. T2.IA^b^ human cells were obtained courtesy of Dr. P. Cresswell, Yale University. *Antibodies*. Mouse IgG was purchased from Jackson laboratories, mouse anti-Ia/peptide monoclonal antibody Y3jP [11] (specific for residues on α-chain of IA^b^ that does not interact with free IA^b^ [38]) and rabbit anti-MHC class II cytoplasmic tail (anti-CII) antibody with specificity for α-chain as well, were a kind gift from Dr. A.Rudensky (Memorial Sloan Kettering Cancer Center, NY).

### Isolation of I-A^b^

I-A^b^ was isolated by affinity chromatography on an mAb Y3jP-sepharose column as previously described [39], [40]. Briefly, purified antibodies (IgG and/or Y3jP) in a coupling buffer (0.2 M NaHCO_3_, 0.5 M NaCl, pH 8.56) were mixed with prewashed CNBr-activated sepharose 4B (GE Healthcare) overnight at 4°C. Excess antibody was washed away with at least 5 volumes of the coupling buffer. The sepharose slurry was resuspended in 0.1 M Tris-HCl, pH 8.0, and incubated for 2 h at RT to block any remaining active groups. The sepharose was washed alternately with acidic and basic buffers for three cycles. Each cycle consisted of a wash with 0.1 M acetate buffer, pH 4.0, containing 0.5M NaCl followed by wash with the coupling buffer at pH 8.56.

A chain of affinity columns (uncoupled Protein A sepharose, mouse IgG-Protein A sepharose, Y3jP-Protein A sepharose) were washed with PBS and equilibrated in NP-40 lysis buffer. Filtered lysate was sequentially run through these columns. Columns were washed with at least 5 column volumes of lysis buffer with 1% octyl-β-D-Glucopyranoside, until OD280 of the wash was equal or less than 0.05U. 750 µl fractions were eluted with the Diethylamine (DEA) buffer [25 mM Diethylamine (Sigma-Aldrich), 1% octyl-β-D-Glucopyranoside (A.G. Scientific, INC), 150 mM NaCl, 25 mM PMSF, 10 mM TLCK, pH 10.5] and neutralized with 150 µl of the 0.5 M Tris pH 6.8. Western blot analysis using rabbit anti-MHC class II cytoplasmic tail (anti-CII) antibody was performed to test each fraction for the presence of MHC class II protein. MHC class II containing fractions were combined for each GILT-WT and/or GILT−/− specimen respectively, dialyzed against mQH_2_O (3×1L), using Spectra/Por3 Dialysis Membrane (Spectrum) with a molecular weight cut-off of 3,500 kDa, and lyophilized overnight.

### Peptide elution

Reverse Phase High Performance Liquid Chromatography (RP-HPLC) purification of I-A^b^ containing GILT-WT and GILT−/− samples was performed on Agilent 1100 Chromatographic system (Agilent Technologies). Chromatographic separation of peptides and I-A^b^ molecules was achieved on Agilent Zorbax 300SB-C_18_ column (4.6 mm ID×150 mm, 5 µm), with an Agilent Zorbax High Pressure, Reliance Cartridge, (4.6 mm ID×12.5 mm) Guard-Column. The aqueous solvent consisted of: 0.1% TFA, 8.7% acetic acid and 2.2% formic acid in HPLC quality water, and the organic solvent consisted of 0.08% TFA in acetonitrile. Flow rate was 1000 µl/min and the gradient was run from 0% organic solvent to 100% organic solvent in 30min. 35×1ml fractions were collected for each GILT-WT and GILT−/− sample.

### Detection of I-A^b^+ fractions and standardization of GILT-WT and GILT−/− samples

I-A^b^ positive fractions were detected by dot blot using rabbit anti-CII (α chain specific) cytoplasmic tail antibody (1∶500 dilution) and goat-anti-rabbit HRP (1∶2500 dilution). Immunoreactive bands were visualized using the enhanced chemiluminescence Western Lightning^tm^ (PerkinElmer) system. I-A^b^ positive fractions (as detected by dot blot) were pooled for the quantification of MHC class II molecules and the rest of the fractions were pooled for mass spectrometry analysis of the MHC class II associated peptides for the GILT-WT sample and the GILT−/− sample, separately. For the quantifications, aliquots of 25 µl, 12.5 µl and 6 µl from the GILT WT and the GILT−/− pooled fractions were separated by SDS-PAGE. Proteins were then transferred onto PVFD membrane and I-A^b^ molecules detected by rabbit anti-CII cytoplasmic tail Ab (1∶500 dilution). IRDye^tm^ 800 goat anti-rabbit secondary antibody (LI-COR Biosciences) was used as the reporter. Bands were scanned using an Odyssey infrared imaging system (LI-COR Biosciences), and analyzed/quantified by the Odyssey 2.1 software (LI-COR Biosciences).

### iTRAQ labeling of peptides (GILT-WT and GILT−/− I-A^b^ negative fractions)

iTRAQ (isobaric tags for relative quantitation) labeling was performed according to the manufacturer's instructions (Applied Biosciences). Two isotopes (114 and 117) from the fourplex (114, 115, 116, and 117 m/z reporter ions) iTRAQ® reagent were used. Due to the isobaric mass design of the iTRAQ reagents, differentially labeled proteins do not differ in mass. Relative quantification of proteins is achieved by comparing peak intensities of reporter ions observed in fragmentation spectra of iTRAQ-labeled peptides. The GILT-WT samples were labeled with the iTRAQ-114, and the GILT−/− samples were labeled with the iTRAQ-117, under identical conditions. These two differently labeled samples (GILT-WT iTRAQ114, and GILT−/− iTRAQ117) were combined right after labeling to ensure identical conditions for the rest of the procedure.

### Ion Exchange Chromatography

5 mM sodium phosphate buffer, pH 3.0, 15% acetonitrile was added to the iTRAQ labeled sample and sample was filtered through a 0.2 µm syringe filter. The samples were applied to the SP-PEEK (SulfoPropyl-Polyetheretherketone) ion exchange column (Waters Corporation) equilibrated with sodium phosphate buffer. Buffer A (5mM sodium phosphate buffer, pH 3.0, 15% acetonitrile) and buffer B (same as buffer A with the addition of 1M NaCl) were used to create a gradient starting from 0 to 40% B (20ml), and to 100% B (5ml). Total of 17 fractions were collected and desalted with MacroSpin™ Columns (The Nest Group, Inc., Southboro, MA).

### Nano-Liquid Chromatography-Electrospray ionization Tandem Mass Spectrometry (Nano-LC-ESI-MS/MS)

Nanoflow chromatography was performed with a Tempo™ nano system (Applied Biosystems). A 5µl sample was injected onto the 75 µm, C18 reverse phase column (Vydac C18), at a flow rate of 200 µl/min, and operating pressure of 1,200 psi. The peptides were eluted using a 5–60% acetonitrile-water gradient over a 120 min period with a flow rate of 300 nl/min, and a total run time of 150 min. Samples from the reverse-phase column were inserted directly into the nano electrospray needle using a QSTAR ELITE Hybrid LC/MS/MS (Applied Biosystems), a high performance quadrupole time-of-flight (QqTOF) mass spectrometer with Analyst® QS 2.0 software. ProteinPilot™ 2.0 software with the Paragon™ Algorithm (Applied Biosystems) was used for post data analysis. The novel Paragon™ Algorithm for database searching can efficiently consider about 150 modifications and unexpected cleavages to identify peptides from MS/MS spectra.

## Supporting Information

Table S1 A large amount of data collected from the mass spectrometer was rapidly processed by analysis software Protein PILOT with the search engine PARAGON [5] to identify peptides sequences of I-Ab-bound peptides which resulted in the detection of more than 5,000 peptide species. Of these, 511 distinct peptides with iTRAQ117 (GILT−/− sample) and/or iTRAQ114 (WT sample) were considered statistically significant based on the confidence score associated with the peptide identification. These peptides are listed in Supplementary Table 1.
(0.11 MB XLS)

Table S2 (0.07 MB DOC)
