# Supplementary material for: Comparative Quantitative Mass Spectrometry Analysis of MHC Class II-Associated Peptides Reveals a Role of GILT in Formation of Self-Peptide Repertoire
Source: PLoS One. 2010 May 12;5(5):e10599. doi: 10.1371/journal.pone.0010599 (PMC2868880; doi:10.1371/journal.pone.0010599)
Supplement: Table S2 — (0.07 MB DOC) [file pone.0010599.s002.doc]

**Supplementary Table 2. Peptides preferentially expressed by MHCII isolated from GILT-/- cells.**

| **Peptide sequence** | **Length** | **Peptide Source** | **Position** | **No of Cys** | **KO:WT 117:114** |
| --- | --- | --- | --- | --- | --- |
| 1. GEPGGAGADGV | 11aa | P08121 Collagen alpha-1 (III) chain precursor [Col3a1] (1464aa) | 743-753 | 21 | 125.99 |
| 2. SSTAPDAAAGG | 11aa | Q6AXD2 Abi2 protein [Abi2] (487aa) | 312-322 | 1 | 65.55 |
| 3. HGASPNTTN Deamidated(N)@6 | 9aa | Q8VC68 Ankyrin 3, epithelial [Ank3] (1726aa) | 438-446 | 20 | 61.00 |
| 4. AATEGTTAT | 9aa | Q6PGB8 Probable global transcription activator SNF2L1 (ATP-dependent helicase SMARCA1) [Smarca1] (1046aa) | 42-50 | 12 | 60.44 |
| 5.MASGVGVGAGLGGGLNQRMDSYAHMNW Oxidation(M)@19 | 28aa | P48432 Transcription factor SOX-2 [Sox2] (319aa) | 141-168 | 1 | 58.64 |
| 6. ATAGSGGVNGG iTRAQ4plex(T)@2 | 9aa | Q00PI9 Heterogeneous nuclear ribonucleoprotein U-like protein 2 (MLF1-associated nuclear protein) [Hnrnpul2] (745aa) | 132-142 | 11 | 53.82 |
| 7. PLSSREGRSRQRPGGSGDRGV Deamidated(R)@5, Deamidated(R)@8, Deamidated(Q)@11, Deamidated(R)@12 | 21aa | XP_001474500.1 Gene info PREDICTED: hypothetical protein (265aa) | 199-219 | 4 | 52.65 |
| 8. GGGGAGGAGGGSGGGGSRAPPEELS Deamidated(R)@18, Oxidation(P)@21 | 25aa | AAH94426.1 Six3 protein (347aa) | 55-79 | 5 | 52.64 |
| 9. CDPALSLKDNGCLIACGSELGT Methylthio(C)@1, iTRAQ4plex(K)@8, Methylthio(C)@12, Methylthio(C)@16 | 22aa | A2AC93-2 Isoform 2 of Dynein intermediate chain 2, axonemal (611aa) | 442-463 | 6 | 50.80 |
| 10. SGAQPGGVPSAPTGPLGPP | 19aa | Q9Z1R2 Large proline-rich protein BAT3 (1154 aa) | 465-483 | 9 | 40.90 |
| 11. GRSAPSDERGGGGRRTAATGV Deamidated(R)@2 | 21aa | XP_001473144.1 Gene info PREDICTED: hypothetical protein (294aa) | 71-91 | 5 | 33.33 |
| 12. SAQVVVGPVSEAEPPKASSA iTRAQ4plex(K)@16, iTRAQ4plex(S)@18 | 20aa | Q9D424 Calcium-binding tyrosine phosphorylation-regulated protein (Calcium-binding protein 86) (453aa) | 239-258 | 4 | 26.50 |
| 13. AYHGLPGGP | 9aa | Q66L40 Slc18a2 protein [Slc18a2] | 392-400 | 19 | 25.64 |
| 14. CASPLITTATFAYWGQGT Methylthio(C)@1 | 18aa | AAX90134.1| immunoglobulin heavy chain (24aa) FRAGMENT | 1-18 | 1 | 17-25 |
| 15. TPQDSRQSIQKPPSPGAEED No iTRAQ4plex(K)@11, Dehydrated(S)@14 | 20aa | Q9EQM5 Homeobox protein GPBOX (Reproductive homeobox 9) (227aa) | 3-22 | 1 | 20.9 |
| 16. EDDIEADHVGTYGISVY | 17 | P14434 HA2B_MOUSE H-2 class II histocompatibility antigen, A-B alpha chain precursor (IAalpha) [H2-Aa] (256aa) | 24-40 | 4 | 10-16 |
| 17. GGGGGGWHSGQRSQQR Deamidated(Q)@14 Oxidation(P)@1 | 16 | A1A542 2310002B14Rik protein (78 aa) | 28-43 | 3 | 10-15.97 |
| 18. HGNGPALGNSSATRWQPPVFPGGRTFGA Oxidation(N)@9, Deamidated(Q)@16 | 28 | Q8CHP0 Zinc finger CCCH domain-containing protein 3 [Zc3h3] (950 aa) | 25-52 | 24 | 14.21 |
| 19. GGAGGHVIGGSRTDQSSGT | 19 | Q8K2R3 Armcx4 protein (Fragment) Armadillo repeat containing, X-linked 4 [Armcx4] (995 aa) | 149-167 | 16 | 9-13.70 |
